# Lyssaviruses and rabies: current conundrums, concerns, contradictions and controversies

**DOI:** 10.12688/f1000research.10416.1

**Published:** 2017-02-23

**Authors:** Charles Rupprecht, Ivan Kuzmin, Francois Meslin

**Affiliations:** 1 Wistar Institute, Philadelphia, PA, 19104, USA; 2 University of Texas Medical Branch at Galveston, Galveston, TX, 77555, USA; 3 DVM, former Team Leader, Neglected Zoonotic Diseases, WHO Headquarters, Geneva, Switzerland

**Keywords:** lyssaviruses, rabies, rabies vaccine, zoonoses

## Abstract

Lyssaviruses are bullet-shaped, single-stranded, negative-sense RNA viruses and the causative agents of the ancient zoonosis rabies. Africa is the likely home to the ancestors of taxa residing within the Genus *Lyssavirus*, Family *Rhabdoviridae*. Diverse lyssaviruses are envisioned as co-evolving with bats, as the ultimate reservoirs, over seemingly millions of years. In terms of relative distribution, overt abundance, and resulting progeny, rabies virus is the most successful lyssavirus species today, but for unknown reasons. All mammals are believed to be susceptible to rabies virus infection. Besides reservoirs among the Chiroptera, meso-carnivores also serve as major historical hosts and are represented among the canids, raccoons, skunks, mongooses, and ferret badgers.  Perpetuating as a disease of nature with the mammalian central nervous system as niche, host breadth alone precludes any candidacy for true eradication. Despite having the highest case fatality of any infectious disease and a burden in excess of or comparative to other major zoonoses, rabies remains neglected. Once illness appears, no treatment is proven to prevent death. Paradoxically, vaccines were developed more than a century ago, but the clear majority of human cases are unvaccinated. Tens of millions of people are exposed to suspect rabid animals and tens of thousands succumb annually, primarily children in developing countries, where canine rabies is enzootic. Rather than culling animal populations, one of the most cost-effective strategies to curbing human fatalities is the mass vaccination of dogs. Building on considerable progress to date, several complementary actions are needed in the near future, including a more harmonized approach to viral taxonomy, enhanced de-centralized laboratory-based surveillance, focal pathogen discovery and characterization, applied pathobiological research for therapeutics, improved estimates of canine populations at risk, actual production of required vaccines and related biologics, strategies to maximize prevention but minimize unnecessary human prophylaxis, and a long-term, realistic plan for sustained global program support to achieve success in disease control, prevention, and elimination.

## Introduction

Rabies is not a simple long-ago vestige, nightmarish myth, or literary allegory but rather a significant viral encephalitis with the highest case fatality of any conventional infectious disease. Who else, besides those afflicted and affected, should care about rabies today? Legions—including the true animal lover, anthropologist, administrator, caver, educator, environmentalist, farmer, medical professional, traveler, health economist, hiker, historian, humanist, industrialist, legislator, modeler, philanthropist, sociologist, student, conservation biologist, and life scientist, to name a few by vocation or avocation—curious for elevated self and situational awareness, caring for the common good, intrigued by this view of life from an applied microbiological and ecological perspective or challenged by the allure for professional intervention in nature, represented by the less-than-apparent “ *non*-low-hanging fruit”.

To simplify the infectious cycle of rabies, exposure is direct, not by environmental deposition, but rather individual-to-individual intra- and inter-specific transmission, usually occurring via bite. Millions of highly neurotropic virions are excreted intermittently in the saliva of a rabid host, days to weeks before overt morbidity and eventual demise, entering the peripheral nervous system of a bite recipient. *In vivo*, from local depots and centripetal transit in the axoplasm, primary replication occurs within neurons of the central nervous system (CNS). Thereafter, centrifugal passage occurs from the CNS to a number of highly innervated sites, including the salivary glands. Oral, mucosal, or transdermal delivery of virions occurs by normal daily mammalian interactions. Failing these routine modus operandi pathways, altered unusual behaviors offer a variety of options for secondary contacts. These may range from mania to paresis and paralysis, with deliberate transmission options of agonistic encounters and biting, increased movement outside of normal home range/territories, or acute death, with predation by others upon virion-laden tissues and organs of the affected host. If a productive infection ensues, the entry-reproduction-exit cycle is poised to begin anew after initiation, taking days, weeks, months, or (rarely) years of incubation before excretion or obvious clinical manifestation. Such obligate, parasitic virions ensure elegant self-transfer by exploitation of the normal through to the bizarre. Relatively distant viral familial relatives hail among invertebrates and plants, but warm-blooded vertebrates are the rabies-prone hosts. Although these agents predated *Homo sapiens*, their current distribution, abundance, and diversity likely exceed pre-historic comparisons, especially mediated during the Anthropocene period.

Among warm-blooded vertebrates, birds are susceptible to infection, but rabies predominates naturally among various mammalian populations. Within the Mammalia, a virtual alphabet soup of cases has been recorded, from the armadillo to the zebra. Rabies is a significant disease of domestic and wild mammals alike, yet its zoonotic aspect is the cause of major historical infamy. Few and privileged were the civilizations that did not describe the ravages of an entity akin to rabies, such that this infection has impacted art, literature, and cultural practices for millennia. By one small measure, during the time taken by a typical reader to peruse this article, more than 1,300 people will have been exposed to rabies virus (RABV). Annually, tens of thousands of persons will succumb, the majority children. Most are poor, have no access to modern medical care, and will die unreported, frequently at home in a rural village. If among other scales—on the basis of disability-adjusted life-year scores or health economic measures—rabies ranks within the top-ten list of neglected viral zoonoses, one would anticipate that the degree of philanthropic input would be roughly equivalent among such pathogens. Unfortunately, such is not the case. For example, several other neglected viral diseases may have a somewhat smaller presumed impact yet receive far greater attention for international support ( Table 1). Not rooted entirely in science, a more holistic transdisciplinary philosophy assists in a better partial understanding of why such biomedical disparities persist between need and assistance, provoking a bootstraps approach in the field out of frustrated necessity in the face of apparent contradictions.

**Table 1.** Comparison of associated health parameters of two vector-borne diseases and human rabies transmitted by dogs ^a^.

	Yellow fever	Japanese encephalitis	Rabies
Disease	Mild to acute viral hemorrhagic syndrome	Mild to severe viral encephalitis	Acute progressive viral encephalitis
Etiology	Flavivirus	Flavivirus	Lyssavirus
Distribution	Endemic in tropics of about 34 African and about 13 Latin American countries	Endemic in about 24 Southeast Asian and Western Pacific countries	Endemic within about 150 developing countries in the Americas, Africa, and Asia
Transmission	Mosquito	Mosquito	Dog bite
Case fatality	About 20–50% in severe cases	About 30%	>99.9%
Burden	About 84,000–170,000 severe cases	About 68,000 cases	>15 million exposures annually
Annual fatalities	About 29,000–60,000 estimated deaths in Africa alone	About 13,600–20,400 estimated deaths	About 25,000–159,000 estimated deaths
Epidemiological occurrence	Sylvatic cycle and urban outbreaks	Major outbreaks about 2–15 years, intensified during the rainy season	Primarily individual human cases in rural, underserved areas
Vaccination	One dose may be effective, with long-term to lifelong immunity in about 99% of people	Primary and booster doses for childhood Expanded Programme on Immunization incorporation	Currently, requires three or more doses in pre- or post-exposure vaccination
Treatment	Supportive care only	Supportive care only	No specific treatment, comfort care only before death
Prospect for elimination	Vaccination protects humans at risk, but Yellow fever cannot be eliminated in nature.	Vaccination protects humans at risk, but Japanese encephalitis is not a candidate for elimination.	Human rabies can be prevented by vaccination and canine rabies can be eliminated by mass dog vaccination.
Global Alliance for Vaccines and Immunization (GAVI) support (by 2015)	>$264 million USD	About $105 million USD forecast for 2015–2020	$0

^a^World Health Organization ( http://www.who.int/mediacentre/en/).

In light of meaningful global action for the public good, at what level should one come to terms with rabies in the 21st century? Management, control, prevention, elimination, eradication, and so on are often freely bandied about together in today’s lexicon of disease deliberations but are not synonymous terms. Unlike smallpox or rinderpest, rabies is not a candidate for actual eradication today, given the extent of host breadth and diversity. However, rabies has at least three major attributes in common with those other two extinct viral pathogens: validated diagnostic protocols, safe and effective vaccines, and the epidemiological insight to apply those laboratory tools and licensed biologics for sound prevention and control practices. Somewhat paradoxically, based upon a century of experience, modern rabies management accomplishes, in a truly One Health capacity, what no other comparable zoonosis program can achieve in tandem: human cases prevented by avoiding defined exposure and seeking prophylaxis after exposure; primary canine and secondary species infections eliminated, by mass immunization; and significantly, wild carnivore viral perpetuation interrupted via oral vaccination efforts on a landscape scale. Building upon such apparent progress, this review aims to provoke renewed discussions on several of the current issues and challenges related to modern lyssavirus taxonomy, phylogeny, surveillance, prevention, treatment, control, and elimination, based in part upon the opinions of the authors, representing more than a century of collective person-years of introspective knowledge, skills, and abilities in the field—not as an historical aside alone, but rather within the context of evidence from the peer-reviewed literature, focusing upon relevant publications primarily within the past few years ^1– 115^. Our hope is, in some small manner, to educate, enlighten, engage, and enable others to participate meaningfully in these remaining endeavors, within the realm of a ‘science of conviction’.

## An evolving viral taxonomy: what is in a name?

Taxonomy, a formal attempt at objective, systematic classification and naming of entities in the complex milieu of life on earth, represented by plants, animals, and so on, also extends to the microbiological arena. Both RABV and a group of phylogenetically related viruses (all of which cause the acute progressive encephalomyelitis known as rabies) belong to the genus *Lyssavirus*, within the family *Rhabdoviridae* and the order Mononegavirales, the single-stranded, non-segmented, negative-sense RNA viruses. With active surveillance and technical advances offered by next-generation sequencing, the taxonomy of the *Rhabdoviridae* is developing with increased complexity and new rhabdoviruses are being characterized ^1^. However, although new lyssaviruses are also being described, the genus forms a well-delineated cluster within the family and does not have any close relationships with other rhabdoviruses.

Recently, the International Committee on Taxonomy of Viruses (ICTV) moved toward a partial appropriation of binomial species nomenclature, which exists in other fields of taxonomy. However, the ICTV applied such naming inversely, so the genus name appears after the species name ^2^. An objective rationale for this change is somewhat difficult to fathom unless the ICTV is trying to underline the fact that “real” virions and the concept of viruses and viral species are totally different entities. Although virions are observed by electron microscopy, no one has ever seen a “virus”. Virions are particulate, whereas viruses are conceptual populations of microorganisms, and more so virus species are “polythetic classes” (or fabricated “containers”) in which certain agents are placed artificially on the basis of their genetic, morphological, and physio-chemical properties ^3^. In other fields of biology, one would use the scientific name of an organism as the synonym for its species, but in virus taxonomy this is not the case. For example, now RABV belongs to the type species of the *Lyssavirus* genus, termed *Rabies lyssavirus*. So speaking in general of the virus as an organism, one still uses the former, whereas the latter is employed in a taxonomic context only. Inherited operational difficulties of use—for example, RABV (a virus) but *Rabies lyssavirus* (a species); Mokola virus (a virus) but *Mokola lyssavirus* (a species) and so on, also noting that virus names, but not species names, can be abbreviated—may lead to further changes of this binomial nomenclature in future years ^4^.

**Figure 1.**
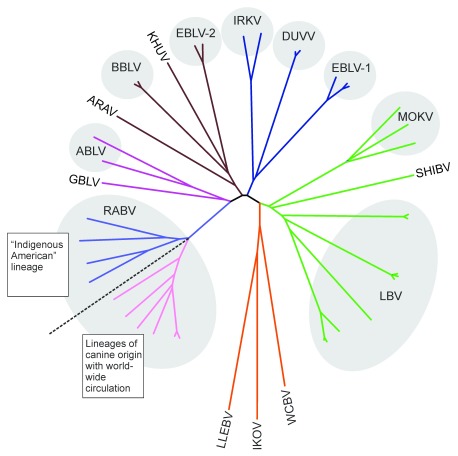
Extant lyssavirus phylogeny. Unrooted phylogenetic tree of currently recognized and putative lyssaviruses (neighbor-joining method, p-distances matrix). Lineage colors correspond to the same lyssaviruses depicted in Figure 2. ABLV, Australian bay lyssavirus; ARAV, Aravan virus; BBLV, Bokeloh bat lyssavirus; DUVV, Duvenhage virus; EBLV-1, EBLV-2, European bat lyssaviruses, type 1 and 2; GBLV, Gannoruwa bat lyssavirus; IKOV, Ikoma lyssavirus; IRKV, Irkut virus; KHUV, Khujand virus; LBV, Lagos bat virus; LLEBV, Lleida bat lyssavirus; MOKV, Mokola virus; RABV, Rabies virus; SHIBV, Shimoni bat virus; WCBV, West Caucasian bat virus.

**Figure 2.**
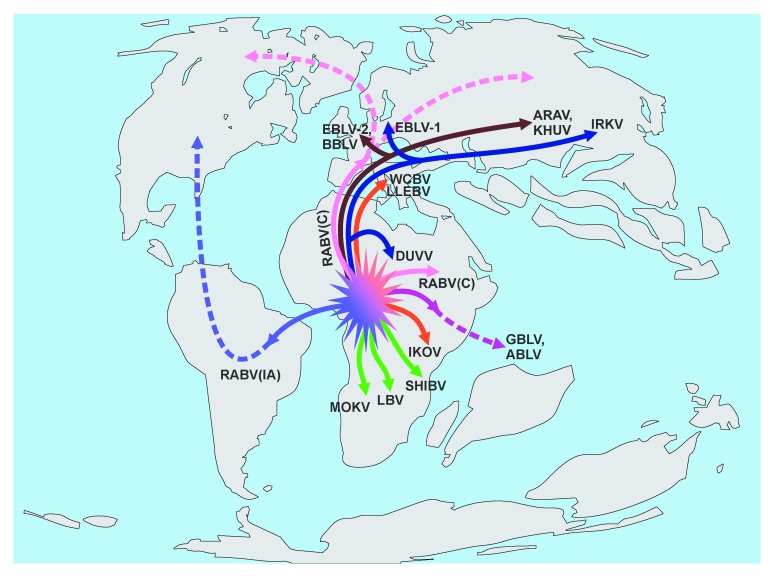
Proposed lyssavirus radiations. Highly speculative schematic depiction of the ancient spread of proto-lyssaviruses on a proposed map of the continents as they were present during the late Cretaceous period. Solid lines show hypothetical directions of lyssavirus ancestor distribution at that time, and dashed lines show further spread thereafter with additional continental drift. Although the “out of Africa” hypothesis dominates the scheme, this does not discount the potential role of Antarctica in biogeographic dispersal with bat-virus links to both Australia and South America as suggested for other pathogens ^93^. ABLV, Australian bay lyssavirus; ARAV, Aravan virus; BBLV, Bokeloh bat lyssavirus; DUVV, Duvenhage virus; EBLV-1, EBLV-2, European bat lyssaviruses, type 1 and 2; GBLV, Gannoruwa bat lyssavirus; IKOV, Ikoma lyssavirus; IRKV, Irkut virus; KHUV, Khujand virus; LBV, Lagos bat virus; LLEBV, Lleida bat lyssavirus; MOKV, Mokola virus; RABV, rabies virus; RABV(IA), rabies virus, “indigenous American” lineage; RABV(C), rabies virus, “carnivore” strain (further shifted to other host mammals); SHIBV, Shimoni bat virus; WCBV, West Caucasian bat virus.

Other species in the genus include *Aravan lyssavirus*, *Australian bat lyssavirus*, *Bokeloh lyssavirus*, *Duvenhage lyssavirus*, *European bat lyssaviruses*, *type 1* and *type 2*, *Ikoma lyssavirus*, *Irkut lyssavirus*, *Khujand lyssavirus*, *Lagos bat lyssavirus*, *Mokola lyssavirus*, *Shimoni bat lyssavirus*, and *West Caucasian bat lyssavirus* ^2^. Two other putative lyssaviruses do not yet have taxonomic status. One is Lleida bat lyssavirus (LLEBV), identified previously by only a partial genome sequence, while isolation attempts continue ^5^. The other, soon to be submitted to the ICTV for review, is the most recently described Gannoruwa bat lyssavirus ^6^. Lyssavirus phylogeny is depicted in Figure 1 (with colors of the branches applicable to Figure 2).

Globally, lyssaviruses have been subdivided into two phylogroups on the basis of genetic distances within their G-protein ectodomains and serologic cross-reactivity ^7^. Phylogroup I includes *Rabies lyssavirus*, *Aravan lyssavirus*, *Australian bat lyssavirus*, *Bokeloh lyssavirus*, *Duvenhage lyssavirus*, *European bat lyssaviruses*, *type 1* and *type 2*, *Irkut lyssavirus*, *Khujand lyssavirus*, and the new Gannoruwa bat lyssavirus. Phylogroup II includes *Lagos bat lyssavirus*, *Mokola lyssavirus*, and *Shimoni bat lyssavirus*. The remaining *West Caucasian bat lyssavirus*, *Ikoma lyssavirus*, and Lleida bat lyssavirus are not included in either phylogroup. Although phylogenetically they do appear related, the amount of genetic divergence and absence of cross-neutralization do not allow placement in a single phylogroup on the basis of existing demarcation criteria ^8^. Genetic distances between lyssaviruses from different species are significantly shorter than those in other rhabdovirus genera. Therefore, other characters, such as antigenic reactivity patterns with monoclonal antibodies and ecologic properties (including distribution and host range), are used imperfectly for demarcation between the viral species ^9^. This demarcation is based on expert opinion of a well-qualified taxonomic study group. With increasing ICTV debate toward unification of virus taxonomy based on genetic distances, in the near future there may be a re-classification attempt, in which all phylogroup I viruses are segregated into one species (for example, *Rabies lyssavirus*?) and all phylogroup II viruses are segregated into another. Of course, such re-classification would miss important characteristics used for species demarcation at present and may have potential socio-economic or bio-political consequences for certain areas. For example, some places where RABV is not thought to circulate, such as in Australia or Western Europe (but where other lyssaviruses are present among bats), might lose their self-defined “rabies-free” status, on the basis of viral taxonomic re-organization, creating greater confusion, with potential public health, veterinary, or economic repercussions, if suddenly recast into the same disease status as Africa, Asia and the New World. Arguably, the term “rabies” appears to garner greater weight and seriousness than the less familiar designation “bat lyssavirus”.

## A conundrum over lyssavirus origins, emergence, perpetuation—and extinctions?

All phylogroup I lyssaviruses circulate in bats. Among these, only RABV is also adapted to perpetuation in carnivores. Within phylogroup II, both Lagos bat virus (LBV) and Shimoni bat virus are bat-borne whereas the reservoir of Mokola virus (MOKV) remains unknown ^10^. MOKV was isolated on a few occasions from shrews and from spill-over infections to cats and a dog ^11^. The West Caucasian bat virus (WCBV) was isolated from a bat, and LLEBV genetic material was identified in a bat as well. Ikoma lyssavirus (IKOV) was isolated from an African civet, but phylogenetic relatedness of this virus to WCBV and LLEBV suggests that it might be a spill-over infection, such that IKOV is actually a bat-borne virus as well ^8^. Enhanced surveillance activities may help to resolve this point.

The evolutionary history of the lyssaviruses is very poorly understood. Common observations include strong purifying selection and neutral evolution of viral genomes ^12– 14^. The substitution rates along lyssavirus genomes are similar between viruses from different species and between different viral genes, approximating 3.8 × 10 ^−5^ to 2.1 × 10 ^−3^ substitutions per site per year ^13, 15, 16^. An episodic diversifying selection was suggested in some studies, under a mixed effects model of episodic (MEME) selection. However, such endeavors failed to identify specific amino acid substitutions involved in host shifts with subsequent virus adaptation to a new host species (with increased fitness and fixation of favorable mutations) and as such should be interpreted cautiously ^15, 16^. This is not too surprising given that only the current sequences (sampled during the past 30 to 40 years from well-established reservoir species) were available for the analysis whereas the natural history of lyssavirus speciation is set in several orders of magnitude longer.

Extant lyssaviruses appear well adapted to their reservoir hosts. Comparison of LBV gene sequences sampled in South Africa over the past several decades revealed over 95% genome conservation ^17^. In another study, the genome of LBV isolated in Kenya was 99% identical to the genome of another LBV isolate obtained in Senegal 22 years earlier ^18^. Recent host shifts might help to elucidate the mechanisms of lyssavirus evolution but are not common. Spill-over infections usually result in dead-end events ^19^. An empirical observation from several short-term host shifts of bat RABV to carnivores suggested that S _242_ substitution in the viral G protein might warrant fitness to bat hosts but that A/T _242_ substitution is typical for carnivore-associated RABVs ^20^. However, no further studies in the development of this hypothesis have been performed.

Early attempts to create a timescale for the natural history of lyssaviruses on the basis of substitution rates (molecular clocks) suggested that the most recent common ancestor (MRCA) of lyssaviruses existed 7,000 to 11,000 years ago but that the MRCA of the present RABV existed 900 to 1,500 years ago ^12^. In line with this, the MRCA age of North American bat RABVs was estimated to be 118 to 233 years ^13^ to 220 to 750 years ^16, 21^.

Other studies that used essentially the same approaches and similar or slightly larger datasets extended the MRCA of bat RABVs to 436 to 1,107 years ^15^ or to 500 years for one of the bat-associated RABV lineages ^22^. However, an increasing amount of evidence suggests that timescale estimation performed on a limited set of recently sampled sequences cannot provide realistic inferences for viruses evolving under constraints of purifying selection ^23^. For example, an alternative approach with improved substitution saturation increased the MRCA of coronaviruses for several orders of magnitude compared with previous molecular clock estimates, and the resulting timescale was comparable to that of the coronavirus reservoir hosts ^24^. In general, evolutionary rates observed in a set of sequences exhibit time dependency and are increased toward the present because of the transient mutations yet to be removed by purifying selection. Therefore, substitution rates estimated during long time frames will be lower systematically than those obtained during short time frames. This effect is as strong as, or stronger than, purifying selection, which shapes virus evolution ^25, 26, 27^. In other words, timescale estimations based on substitution rates are useful only for the time frame encompassed by the sampling period but cannot be extrapolated easily for longer periods of time.

Present distributions, diversity and compartmentalization of lyssaviruses, and the empiric observation of viral genome conservation reveal great contrast to the suggested recent age of their MRCA. If one entertains that the published suggested timescale estimates of lyssaviruses are fallacious, restricted by analysis of limited sets of available isolates, one can use several other lines of evidence to hypothesize their origin and longer-term evolution. All the diversity of lyssaviruses is represented in bats (except for MOKV and IKOV, for which the reservoir hosts are yet to be established definitively), whereas carnivores maintain perpetuation of several lineages of RABV only. As such, it is commonly accepted that bats are the primary evolutionary hosts of lyssaviruses ^8, 12, 28^. Lyssaviruses of multiple species (with the notable exception of RABV) circulate in Old World bats, but only RABV circulates in New World bats. Furthermore, RABV lineages present in New World bats, along with a few lineages circulating in skunks and raccoons (the so-called “indigenous American” RABV), are paraphyletic to the RABV lineages circulating in carnivores in the Old World and to the “cosmopolitan” canine lineage, which (as believed) was dispersed worldwide with human migrations across the globe ^29^. Nevertheless, all RABV lineages are monophyletic when compared with lyssaviruses of other species and demonstrate a greater sequence identity between each other than to lyssaviruses of other species ^30^. From this evidence, it is tempting to speculate that an ancient RABV ancestor (already distinct from lyssaviruses of other species) circulated in early bats in the territory of Gondwana, as bats presumably originated in the region of modern Africa ^31^. A bat morphologically similar to extant bats, *Onychonycteris*, lived about 52.5 million years ago ^32^. Other related species might have existed earlier. Molecular inferences suggested that the MRCA of present bats existed at least 80 million years ago, potentially pushing bat origins into the Cretaceous period ^31, 33^. Such an ancient chiropteran origin of RABV might explain the existence of the independently evolved “indigenous American” virus lineage and the absence of similar RABVs in related bat species between the Americas and the Old World (for example, in *Myotis* spp. and *Eptesicus* spp.). South America and Africa split from each other between 100 and 84 million years ago and were separated by the South Atlantic Ocean. Ancient bats are hypothesized to have spread from Africa to South America slightly later, via island hopping or direct intercontinental flight ^31^. Perhaps, at that time, bats were already infected with a progenitor of the “indigenous American” RABV ( Figure 2). This virus might be well adapted to bat hosts (for example, owing to substitutions such as S _242_ in the viral G protein) and compartmentalized in numerous bat species, such as the indigenous phyllostomatids, across the Americas, with a later host shift to other bat species, skunks, and raccoons. Phylogenetic analysis suggested rapid diversification of bats, with all families having evolved before the late Eocene epoch ^31^, and the same might be true for the viruses that co-evolved with their hosts, followed by millions of years characterized by stasis and slow genetic drift under constraints of purifying selection. Possibly, RABV lineages that continued to evolve in the Old World did not obtain such favorable substitutions and could not colonize multiple bat species as in the Americas but rather switched to carnivores as a comparatively recent event. Of course, this hypothesis does not explain the absence of RABV in Old World bats. However, given the less-than-ideal level of surveillance in the Old World tropics, it is possible that bat RABV does exist there but is undiscovered. Considering that four new lyssaviruses were described during the last decade (including Bokeloh bat lyssavirus in Western Europe, where surveillance is quite adequate) and that the most recent of these, Gannoruwa bat lyssavirus, was described in Sri Lanka in 2016 only ^6^, one should expect further discoveries of lyssaviruses in the Old World. The absence of non-RABV lyssaviruses in the Americas can be explained at least in part by the fact that even at present these viruses have a limited geographic distribution and host ranges compared with RABV. If these characteristics were similar in the past, such viruses might not spread to the territory of the present Americas once Africa and South America drifted further apart ( Figure 2).

Alternatively, one can speculate that many viruses (including multiple bat RABVs in the Old World and non-RABV lyssaviruses in the New World) became extinct during the Cretaceous-Paleocene event 65 million years ago which, as estimated, wiped out 75% of all biologic species ^34^. As major reservoirs went extinct, so too the viral species adapted to them. Though lacking to date for lyssaviruses, progress in the field of paleovirology and applications to other members of the Mononegavirales support the contention of time scales for some ancient viruses in excess of tens of millions of years in age, co-evolving with their hosts ^35^.

## Neglect of laboratory-based surveillance systems

In an applied One Health context, rabies diagnosis is the only routine procedure applied to a suspect animal that will directly determine the need for specific, life-saving medical intervention in a human at risk ( http://www.cdc.gov/rabies/pdf/rabiesdfaspv2.pdf). Laboratory diagnosis is critical to confirm the status of a suspect case, in part, to justify prophylaxis in exposed persons or animals, to measure objectively the impact of disease prevention programs, and to support certification of a country as free of disease.

Sensitive, specific, economical, and timely diagnostic tests have been available for more than 50 years, and there has been increasing augmentation by molecular methods for routine rabies diagnosis ^10, 36, 37^. Yet despite the innumerable rabies cases that occur in wildlife, domestic animals, and humans on a daily basis, only a very small fraction are diagnosed. Few resources are provided, because no cases are diagnosed, owing to a lack of support, producing a cycle of neglect that minimizes the true understanding of disease burden. Those at highest risk are often of lowest priority, such as the poor, the disenfranchised, and the non-agricultural commodities represented by free-ranging wildlife or community dogs.

Misdiagnosis may result with the presentation of fever and coma in children because of confusion with other diseases, such as cerebral malaria ^38^. Conversely, in some situations, a history of animal bite may be missing because the patient does not realize that exposure has occurred, such that rabies does not enter the original differential diagnosis of encephalitis ^39^. The relationship between exposure and illness may be forgotten, and patient recall may be disconnected because of the lag from the incubation period, which can extend beyond weeks to months or even years ^40^. Moreover, the national laboratory may be located centrally in an urban capital, far from case occurrence in rural areas.

The consequences of neglect are obvious. For example, when juveniles contact rabid puppies, the diagnostic event is missed, the prophylaxis opportunity is gone, and the child dies ^41^. If rabies is not suspected in an infected donor, organs may be transplanted from a patient that dies acutely, producing additional fatal cases in the recipients ^42^. Beyond the individual, at a global level, the frequent lack of inclusion of wildlife in surveillance efforts may mean the difference between a misjudged, rabies-free locality and a newly appreciated enzootic area ^43^. Similarly, translocation of a rabid dog from a canine-enzootic area can threaten the status of another area that has eliminated canine RABV circulation, after great cost and years of effort ^44^. Such reports of importation are not uncommon in the literature, online sources such as ProMED, or communication in the daily news ( http://www.animals24-7.org/2014/06/08/dog-meat-traffic-still-spreads-rabies-in-vietnam/).

Although it is highly desirable to document the presence of new lyssaviruses during pathogen discovery, the basic surveillance information needed to prevent and control what is already known about RABV and its impact upon public health, agriculture, and conservation biology, using available, practical diagnostic tools, is more important ( Table 2).

**Table 2.** Problems and options for improved lyssavirus laboratory-based surveillance.

Apparent issues	Proposed resolution	References
Few reported suspect cases	Improved enhanced or active surveillance	94
Confusion over which test to use for primary case confirmation	Direct fluorescent antibody test implementation on central nervous system (CNS) tissue (gold standard)	10
Limited budget for fluorescent microscopic equipment	Direct rapid immunohistochemistry test	95
Biosafety concerns of animal CNS removal	Focus upon brainstem collection	96
Few trained field staff for de-centralized surveys	Linear flow assay (LFA) screening with confirmatory testing	97
Insensitivity of existing LFA tests	Improved commercial lot release and licensing	98
Need for basic virus variant identification	Monoclonal antibody typing	99
Cultural sensitivity over human autopsy performance	Antemortem collection of skin biopsy, serum, cerebrospinal fluid (CSF), and saliva swabs	100
Requirement for rapid, sensitive, and specific identification of suspect human cases	Real-time polymerase chain reaction of human saliva and biopsy samples	101
Inability for virus neutralization tests of clinical samples	Enzyme-linked immunosorbent assay-based tests of serum and CSF	102
Desire for greater epidemiological investigation of confirmed cases	Whole genome sequencing	103
Need for deep identification of viral quasispecies	Next-generation sequencing	104

## Prophylaxis concerns

Often forgotten, rabies qualifies as a vaccine-preventable disease. Effective rabies biologics have been available for over a century ^45^. Pre-exposure vaccination is highly efficacious for those at risk of exposure, such as veterinarians, laboratory workers, and certain travelers ^46, 47^. After exposure, the prompt and proper application of post-exposure prophylaxis (PEP)—consisting of wound care, infiltration of rabies immune globulin (RIG), and administration of modern rabies vaccines—virtually ensures survival ^48^. Nevertheless, the majority of persons at risk do not receive pre-exposure vaccination and most RABV-exposed patients are never provided adequate PEP. This discrepancy between Advisory Committee on Immunization Practices/World Health Organization (ACIP/WHO) recommendations and reality has sparked strong debate and translational research for more novel, less expensive products; simplified schedules; and improved ease of use ^49– 58^. Outstanding questions for resolution include the following: Can PEP be performed safely without RIG (given that limited availability means more than 90% of persons do not receive it)? Are heterologous (for example, equine) RIG products equivalent to human RIG in regard to safety and effectiveness? If so, how should their production be increased? Is the dose of RIG critical in terms of international units per kilograms or is local infiltration more important, regardless of absolute dose? Does administration of vaccine directly into exposure sites improve survivorship if RIG is not available? Could PEP completion be reduced to one week or less? What is the role for rabies pre-exposure vaccine inclusion into childhood schedules? When will GAVI add human rabies vaccine to its portfolio? Will monoclonal antibodies help to resolve the issues with RIG availability and distribution? Should new biologics be developed to increase the breadth of reactivity against non-phylogroup I lyssaviruses? Is there a role for new adjuvants or attenuated RABV vaccines to obviate the need for RIG? Should genes involved in the innate immune response (for example, interferons) be incorporated into genomes of recombinant RABV vaccine strains? Although the field will always profit from relevant advances, much attention is often spent on academic research on new biologics and less on translational research for the appropriate use of existing products and protocols to prevent rabies now ( Table 3).

**Table 3.** Risk assessment to maximize the utility of rabies prophylaxis after human exposure ^10, 48, 75^.

Category	Issue	Outcome
Species	Is the mammal a reservoir?	Non-reservoirs or non-vectors are less likely to be rabid.
Exposure	Was the exposure due to a bite?	Non-bite exposures are less likely to cause rabies.
Health	Does the animal show compatible clinical signs with an encephalitis or other behavioral abnormalities suggestive of rabies?	In general, apparently healthy animals (even near the end of the incubation period) are less likely than ill animals to excrete virus.
Epidemiological status	What is the occurrence of rabies in the area?	Unless an epizootic or enzootic status is apparent, rabies is less frequent in areas without cases for several years, assuming adequate laboratory-based surveillance.
Event circumstances	Was the exposure provoked?	Often animals may bite if provoked (for example, protecting young, sleeping, or eating).
Observation	Can the dog, cat, or ferret be observed?	If the animal stays apparently healthy during at least 10 days after the bite, no post-exposure prophylaxis (PEP) is needed (or initiated PEP can be discontinued).
Vaccination status	Is the animal up to date on rabies vaccination?	Vaccine failures are possible, but rare, with modern veterinary biologics.
Diagnosis	Is the brain available for a timely examination?	If no rabies virus antigens are detected by a qualified laboratory using an approved test, no PEP is needed.
First aid	Were all wounds washed well?	Proper cleansing with soap and water reduces the viral load in local wounds.
Injury	Does the injury require sutures?	If at all practical, suturing should be postponed, to avoid the opportunity to further contaminate the locality and drive virus deeper in tissues.
Biologics	Are modern vaccines and rabies immune globulin (RIG) available?	If the diagnosis is positive or (under the worst conditions when rabies is strongly suspected) if an unprovoked bite is from an ill, unavailable dog or other reservoir in an enzootic area, begin PEP per current World Health Organization recommendations to the extent possible or support transport to the nearest suitable facility immediately.
Patient health	Is the person immune-competent?	In the severely immune-suppressed patient, such as someone with AIDS, passive immunization with rabies immune globulin (RIG) becomes an even more critical part of PEP.

## Controversy on the horizon: a paradigm shift to treating the “incurable wound”?

Today, as in the past, a diagnosis of a rabies case is, in a statistical sense (>99.9%), synonymous with fatality. There is no proven treatment once clinical signs appear. This presents a huge dilemma to the clinician faced with a rabies patient, with few options ^59^. For a veterinarian, the animal is euthanized. For a physician, the patient may be placed in isolation, and at a minimum palliative care needs to be provided.

Alternatively, the person may be sent home to die. Cumulatively, since the 1970s, reports of very rare, non-fatal human cases (most with a history of vaccination) and the occasional spontaneous “survivor” during animal rabies studies suggested that clinical rabies may not always end in death, prompting the administration of aggressive anti-viral therapy ^60^. As a case in point, in 2004, a Wisconsin teenager bitten by a bat became the first unvaccinated survivor after intensive medical care, coma induction, and administration of anti-viral drugs ^61^. Over the next decade, few additional survivors who received the “Milwaukee protocol” were added to the registry ( http://www.mcw.edu/Pediatrics/InfectiousDiseases/PatientCare/Rabies.htm). Nevertheless, the drive to act is understandable; such undertakings are compassionate and heroic attempts at treating the incurable. Who can witness the face of pathos in a Manila rabies ward and not be sensitized ( http://www.aljazeera.com/programmes/lifelines/2013/09/rabies-lifelines-isolation-patient-2013923164345829402.html)? Who can listen to the heart-breaking personal impact of rabies on a family and not feel moved ( https://www.youtube.com/watch?v=u8o1tOuyghk)? Who can watch “The Girl Who Survived Rabies” and not feel compelled ( https://www.youtube.com/watch?v=qdPuXHhEwDk)? Although there has been sharp debate over the dangers of therapeutic coma and the merits of the Milwaukee protocol, the often-heated discussion has reinvigorated the field in a direction toward consideration of potential treatment administered to the patient with rabies ^62^. Asklepios is but one example of such a renaissance by a consortium of scientists whose focal research aims include the following: the identification of drugs that would inhibit RABV replication, the testing of molecules that may minimize detrimental host responses during RABV infection, the determination of whether alteration of the blood-brain barrier (BBB) permeability could improve treatment effectiveness, and validation of the potential success for such an approach *in vivo* ( http://asklepiosfp7.eu/). Regardless of technical insights, required expertise, the associated expense and related ethics of effort issues, there is also the prospect of survivorship with sequelae and the need for lengthy, potentially lifelong, rehabilitation and quality-of-life considerations ^63^. To make progress, whether in veterinary or human medicine, a more complete approach to rabies treatment should be taken, combining insights on rabies pathobiology gleaned from both experimental animal research and individual human case studies ^64^. Excellent clinical care by a dream team of specialists, anti-viral drugs incorporated from other RNA virus research, targeted immune modulation, the tincture of time, and a healthy dose of luck for appreciable self-cure in a patient with the ideal age, genetic pre-disposition, and clinical staging parameters may offer future hope to a more prescriptive treatment of rabies ( Table 4).

**Table 4.** Consideration for a strategic combination approach to the management and treatment of clinical rabies.

Proposed need	Suggested consideration	References
Management of the dying rabies patient?	Responsible palliative care, toward death with dignity	105
Intensive care of acute progressive rabies encephalitis?	Ventilation, sedation, cardiac monitoring, body temperature regulation, parenteral nutrition, management of vasospasm, and so on	106
Real-time diagnostic support?	Rapid antemortem confirmation, viral characterization, and continued patient monitoring, including serology, amplicons, antigens, and so on	107
Active immunization?	Recombinant vaccines	108
Passive immunization?	Rabies immune globulin or monoclonal antibodies	109
Administration of immunostimulatory oligonucleotides?	Use of PyNTTTTGT compounds, such as IMT504	110
Anti-viral drugs?	Use of known ssRNA virus inhibitors, such as favipiravir (T-705)	111
Targeted host-catalyzed biochemical pathways?	Selection of specific small-molecular-weight compounds	112
Blood-brain barrier permeability enhancement?	Induction of pro-inflammatory chemokines and cytokines	113
Associated pathological decrease of dopaminergic and serotoninergic neurotransmission?	Supplementation with biotin	114
Mitochondrial dysfunction and degenerative changes in neuronal processes?	Relief of potential oxidative stress	115

## Concerns over vaccine needs to achieve global human and dog rabies elimination—by 2030

Throughout the late-20th and early-21st centuries, target dates for the elimination of human-dog-mediated rabies were set by the WHO, the Pan American Health Organization, and other international governmental and non-governmental organizations for Africa, Asia, and Latin America ( 65– 68 http://www.paho.org/hq/index.php?option=com_content&view=article&id=11243%3Astep-up-action-toward-rabieselimination&Itemid=1926&lang=en). These recent end dates were endorsed by the World Health Assembly in May 2013 ^69^.

The main components of this human rabies elimination program are control, prevention, and eventual elimination of rabies in dogs by mass immunization. Successive annual vaccination campaigns, reaching at least 70% of the dog population, led to progressive disease control and ultimately elimination in both dogs and humans ^10, 68^. Estimating the number of canine vaccine doses needed by country, WHO region, and finally globally over time to achieve these goals is of upmost importance. Understandably, dog rabies vaccine production and access have not received the same attention as human rabies biologics, even though the latter are still in somewhat limited availability, particularly in rural areas. Today, overall dog vaccination coverage is estimated to be less than 20% in canine rabies-endemic countries outside of the Americas ( Table 5).

**Table 5a.** Estimated dog populations, annual dog vaccine coverage and number of dog vaccine doses used per WHO region, including China (WPR) and India (SEAR).

Elimination target date	WHO region	Estimated dog population ( Table 6)	Estimated vaccination coverage (see source below)	Dog vaccine doses used annually (in millions of doses)	Percentage of total vaccine doses applied
2020	EUR (Eurasia)	85,612,000	22%	19	24.5
	EMR	26,547,000	32%	8.5	11
	SEAR, without India	72,631,000	20%	14.8	19.1
	India (72% rural)	38,109,000	15%	5.8	7.5
	WPR, without China	38,847,000	19%	7.3	9.4
	China (55% rural)	71,785,000	14%	10	13
	Totals for EMR, EUR, SEAR, and WPR	333,531,000	20%	65.4	84.5
2030	AFR total	77,417,000	16%	12	15.5
	Grand total WHO regions (but AMR)	411,000,000	20%	77.4	100

AFR, African Region; AMR, Region of the Americas; EMR, East Mediterranean Region; EUR, European Region; SEAR, South East Asia Region; WHO, World Health Organization; WPR, West Pacific Region.

**Table 5b.** 

Region	Supportive notes and references
Eurasia (EUR)	22% ^70^
Eastern Mediterranean Region (EMR)	32% ^70^
South East Asia Region (SEAR), without India	Sum of number of dogs vaccinated in SEAR countries belonging to clusters Asia 2, 3, and 4 plus Indonesia according to estimated vaccination coverages (respectively 9, 5, 36, and 24% in 70) divided by an estimated total dog population of about 72.6 million
India	15% (from 70) applied to estimated rural dog population of India ( Table 6)
West Pacific Region (WPR), without China	Sum of number of dogs vaccinated in WPR countries belonging to clusters Asia 2, 3, and 4 plus Mongolia according to vaccination coverage (5.9% from 70) divided by estimated total dog population of 38.8 million ( Table 6)
China	Coverage 14% ^70^ applied to estimated rural dog population of China ( Table 6)
African region	North Africa dog immunization coverage 10%, Southern Africa Development Community (SADC) coverage 23%, West Africa coverage 10%, and rest of Africa 9% ^70^ applied to estimated dog population of relevant geographical clusters ( Table 6), giving for North Africa: 0.7 million dogs vaccinated, for SADC: 7 million, West Africa: 2 million and rest of Africa: 2.5 million. Total Africa: 12.2 million. Dog vaccination coverage Africa: 16%

**Table 6.** Numbers of dogs per human and estimated dog populations per geographical areas, countries and World Health Organization regions.

Geographic area	WHO region	Country	Human population (estimated)	Percentage rural	Human/ dog ratio	Dog population (estimated)
Asia and Eurasian						
Asia 2	WPR	Cambodia	15,135,169	80%	6.60	2,293,207
	WPR	Laos	6,769,727	66%	6.60	1,025,716
	SEAR	North Korea	24,895,480	40%	6.60	3,772,042
	WPR	South Korea	50,219,669	17%	6.60	7,609,041
	SEAR	Myanmar	53,259,018	63%	6.60	8,069,548
	WPR	Vietnam	89,708,900	69%	6.60	13,592,258
Asia 3	SEAR	Bangladesh	156,594,962	72%	14.70	10,652,719
	SEAR	Bhutan	753,947	64%	14.70	51,289
	EMR	Pakistan	182,142,594	64%	14.70	12,390,653
	SEAR	Nepal	27,797,457	83%	14.70	1,890,983
Asia 4	WPR	Philippines	98,393,574	51%	7.00	14,056,225
	SEAR	Sri Lanka	20,483,000	85%	7.00	2,926,143
	SEAR	Thailand	67,010,502	66%	7.00	9,572,929
	Total Asia 2, 3, 4					87,902,753
Other Asia	SEAR	Indonesia	249,865,631	49%	7.00	35,695,090
	WPR	Mongolia	2,839,073	31%	10.5	270,388
	SEAR	India	1,252,139,596	72%	23.0	38,108,596a
	WPR	China	1,357,380,000	55%	10.4	71,784,519 ^a^
	Total other Asia					145,858,593
	Total Asia excluding Pakistan.					**221,370,693**
Eurasia						
	EUR	Kazakhstan				
	EUR	Kyrgyzstan				
	EUR	Russian Federation				
	EUR	Turkmenistan				
	EUR	Tajikistan				
	EUR	Uzbekistan				
	Total Eurasia		898,926,561		10.5	**85,612,053**
**Africa**						
**SADC**					
	AFR	Democratic Republic of the Congo	67,513,677	66%	9.5	
	AFR	Angola	21,471,618	41%	9.5	
	AFR	Zambia	14,538,640		9.5	
	AFR	Malawi	16,362,567		9.5	
	AFR	Tanzania	49,253,126		9.5	
	AFR	Mozambique	25,833,752		9.5	
	AFR	Zimbabwe	14,149,648		9.5	
	AFR	Botswana	2,021,144		9.5	
	AFR	Namibia	2,303,315		9.5	
	AFR	South Africa	52,981,991		9.5	
	AFR	Swaziland	1,249,514		9.5	
	AFR	Madagascar	22,924,851		9.5	
	AFR	Lesotho	2,074,465		9.5	
	Total SADC		**292,678,308**		**9.5**	**30,808,243**
**North Africa**						
	EMR	Morocco	33,008,150		31.2	
	EMR	Algeria	39,208,194		31.2	
	EMR	Tunesia	10,886,500		31.2	
	EMR	Libya	6,201,521		31.2	
	EMR	Egypt	82,056,378		31.2	
	EMR	Western Sahara	500,000		31.2	
	EMR	Sudan (+ south)	37,964,306		31.2	
	Total North Africa		**209,825,049**		**31.2**	**6,725,162**
**West Africa**						
	AFR	Benin	10,323,474		16.8	
	AFR	Burkina Faso	16,934,839		16.8	
	AFR	Cape Verde	498,897		16.8	
	AFR	Ivory Coast	20,316,086		16.8	
	AFR	Gambia	1,849,285		16.8	
	AFR	Ghana	25,904,598		16.8	
	AFR	Guinea	11,745,189		16.8	
	AFR	Guinea-Bissau	1,704,255		16.8	
	AFR	Liberia	4,294,077		16.8	
	AFR	Mali	15,301,650		16.8	
	AFR	Niger	17,831,270		16,8	
	AFR	Nigeria	173,615,345		16.8	
	AFR	Senegal	14,133,280		16.8	
	AFR	Sierra Leone	6,092,075		16.8	
	AFR	Togo	6,816,982		16.8	
	Total West Africa		**327,361,302**	****	**16.8**	**19,485,792**
**Other Africa**						
	AFR	Uganda	37,578,876		9.5	3,955,671
	AFR	Gabon	1,671,711		16.8	99,507
	AFR	Nigeria	173,615,345		16.8	10,334,247
	AFR	Equatorial Guinea	757,014		16.8	45,060
	AFR	Chad	12,825,314		16.8	763,412
	AFR	Rwanda	11,776,522		9.5	1,239,634
	AFR	Burundi	10,162,532		16.8	604,913
	AFR	Republic of Congo	4,447,632		9.5	468,172
	AFR	Ethiopia	94,100,756		31.2	3,016,050
	AFR	Mauretania	3,889,880		31.2	124,676
	AFR	Kenya	44,353,691		9.5	4,668,810
	EMR	Somalia	10,495,583		31.2	336,397
	AFR	Central African Republic	4,616,417		16.8	274,787
	AFR	Eritrea	6,333,135		31.2	202,985
	AFR	Cameroon	22,253,959		16.8	1,324,640
	Total other Africa					**27,458,959**
**Total Africa** **excluding** **North** **Africa and** **Somalia**						77,416,599
**Other** **EMRO**						
	EMR	Afghanistan	30,551,674		35.4	863,042
	EMR	Bahrain	1,332,171		35.4	37,632
	EMR	Djibouti	872,932		35.4	24,659
	EMR	Iran	77,447,168		35.4	2,187,773
	EMR	Iraq	33,417,476		35.4	943,996
	EMR	Jordan	6,459,000		35.4	182,458
	EMR	Kuwait	3,368,572		35.4	95,157
	EMR	Lebanon	4,467,390		35.4	126,197
	EMR	Palestine	4,169,506		35.4	117,783
	EMR	Oman	3,632,444		35.4	102,611
	EMR	Saudi Arabia	28,828,870		35.4	814,375
	EMR	Syrian Arab Republic	22,845,550		35.4	645,355
	EMR	United Arab Emirates	9,346,129		35.4	264,015
	EMR	Yemen	24,407,381		35.4	689,474
	Total EMR including North Africa, Pakistan, Somalia and other EMR countries					**26,546,739**
**Grand total**						**410,946,084**

Adapted from K. Hampson (personal communication, 2014) using World population statistics 2010 at www.worldpopulationstatistiscs.com and expanding on the list of countries eligible for GAVI support, 2014 ( www.gavi.org/supportapply/countries-eligible-for-support/). AFR, African region; EMR, East Mediterranean region; EMRO, Eastern Mediterranean Regional Office; EUR, European (Asian portion) Region; SADC, Southern Africa Development Community; SEAR, South East Asia Region; WHO, World Health Organization; WPR, West Pacific Region.

There is relatively little production, technology development or transfer, import, and subsequent use of canine vaccines in dog rabies-enzootic countries. To prevent rabies in humans, manufacturers from the Northern Hemisphere and an increasing number of producers from emerging markets (for example, China and India) produce enough vaccine annually to deliver approximately 28 million rabies PEP courses in dog rabies-enzootic countries of Africa, Asia, and the Eastern Mediterranean region, preventing nearly 98% of human rabies deaths. Unfortunately, easier access to rabies vaccine, particularly in urban centers of Africa and Asia, has been accompanied by an increasing proportion of PEP (sometimes up to 70%) administered to people who are not at high risk of developing rabies, necessitating greater education and outreach to the public and professionals alike ^70^. Such issues are not uncommon in Europe and North America, where human rabies caused by dogs has been eliminated.

A canine rabies vaccine bank was established in 2012 by the World Animal Health Organization (OIE), with multi-donor support to assist Asian countries initiating (for example, Afghanistan, Laos, and Myanmar) or strengthening (for example, the Philippines, Sri Lanka, and Vietnam) their immunization campaigns ^71, 72^. Though certainly useful, the quantities provided (about 3.7 million doses from 2012 to April 2015) have remained limited in comparison with annual requirements of full-fledged national programs in 10 Asian beneficiary countries. For example, over 3 years, Vietnam received a total of about 1 million doses, but nearly 10 million local dogs need to be vaccinated annually. Similarly, the WHO has expressed an intention of constructing a human rabies vaccine stockpile to rapidly provide quality-ensured vaccines upon request ^73^. Globally, human rabies vaccine quantities produced seem close to adequate, but at a country level, major challenges include the following: assessing unnecessary or inappropriately applied PEP, particularly in urban centers; bringing appropriately staffed “bite treatment centers” to needed communities; providing free or low-cost rabies biologics; and applying economical and easier-to-use PEP regimens to exposed patients ^56^.

A meeting was held at the WHO in 2015 on human and dog rabies vaccines and RIG. Objectives were to discuss forecasting needs, vaccine and RIG quality, and funding and procurement issues, and to estimate quantities of human and dog vaccines needed in the medium and long term at both the country and regional level ^74^. During this meeting, manufacturers of canine rabies vaccines stated that production capacity shortages were not an issue and that output could be increased easily should accurate, financially supported, medium-term vaccine forecasts become available. However, current annual production capacities of major manufacturers are estimated to be near 100 million doses, and maximum capacity is about 150 million doses ^75^. This rabies vaccine production is meant to satisfy the most profitable Northern Hemisphere (for example, USA, Canada, and Western and Eastern Europe) markets, where there are about 170 million owned dogs, whereas only a small share of the 411 million owned and community dogs are vaccinated each year in the defined WHO African, Asian, Eurasian, and Middle Eastern regions ( Table 5). The vaccine requirements of Latin American countries, where dog-mediated human rabies has almost been eliminated (mostly through dog rabies immunization), are estimated at about 42 million doses annually and often are covered largely by local manufacturers based in Mexico and Brazil ^76, 77^. Together, dog rabies-enzootic countries of Central Asia belonging to the European (EU), South-East-Asian (SEA), and Western Pacific (WP) WHO regions account in nearly equal proportions for almost 74% of the total number of canine vaccine doses used. Africa and Eastern Mediterranean regions account for the remaining 15% and 11%, respectively.

The number of vaccine doses needed in canine rabies-enzootic countries of the defined WHO Africa, Asia, Eurasia, and East Mediterranean regions were estimated in the medium (2015–2020) and longer (2020–2029) terms to achieve the goal of human and dog rabies elimination. As a given, these vaccines should comply with all international norms and standards ^10, 37, 78^. In the computing of estimates, one of the most sensitive figures is the human/dog ratio by geographical areas/WHO regions and large countries (China and India). Numbers of dogs per human and country clustering have been used from earlier publications ^70, 79^. Some dog rabies-enzootic countries were added to existing lists. When country-specific data were unavailable, the average estimate from countries within the cluster was applied. Table 6 provides a list of more than 85 dog rabies-enzootic countries with their human and dog populations and dogs-per-human ratio in Africa, Asia, and East Mediterranean regions on the basis of prior work ^70, 80, 81^.

Using the above population estimates to compute canine vaccine needs over time, we used a conservative 10-year country-based dog rabies elimination model divided into a 5-year attack phase, a 3-year consolidation phase, and a 2-year maintenance phase (with vaccination coverages decreasing from 70% to 20% and finally to 5% as the program moved from one phase to another). This model is based on both theoretical and field experiences ^82– 86^. Understandably, national programs will progress at various paces. Some may need shorter or longer attack, consolidation, or maintenance phases than others. Ten years of sustained dog rabies immunization activities were considered long enough to bring human rabies cases down toward zero in most places, if carried out with the joint leadership of the countries and major international organizations ^87^.

Dog vaccine requirements per country were aggregated by WHO regions and time periods. The results of estimated dog vaccine requirements by WHO regions and large countries (for example, China and India) are presented in Table 7. For the European (Eurasia cluster), East Mediterranean, Asian, and Western Pacific WHO regions, the attack phase extends from 2015 to 2019; the consolidation phase from 2020 to 2022; and the maintenance phase from 2023 to 2024, as the target date for human rabies elimination here is 2020. Many countries in the above listed WHO regions particularly the Southeast Asia and Western Pacific regions which carry a large part of the global human rabies burden have committed themselves to achieve the goal of human rabies elimination by 2020 and already scaled-up their dog and human rabies control activities (e.g Philippines, Sri Lanka, Thailand, Vietnam and parts of Indonesia, India & China). For the African Region, the attack phase extends from 2020 to 2024; the consolidation phase from 2025 to 2027; and the maintenance phase from 2028 to 2029, as the target date for human rabies elimination here is 2030. The African region particularly sub-Saharan Africa will benefit from the full 10 year program plus preparatory time as the number of countries with experience of large-scale dog rabies immunization campaigns is comparatively small.

**Table 7.** Estimated dog vaccine requirements in 2014–2030 to achieve human and dog rabies elimination target dates in World Health Organization regions.

Program phase	Attack phase (5 years)	Consolidation phase (3 years)	Totals (phases 1 and 2)	Maintenance phase (2 years)	Total (phases 1, 2, and 3)	Number of dogs vaccinated per region in percentage total	Grand total
Time lines	2015–2019	2020–2022	2015–2022	2023–2024	2015–2024	2015–2024	2015–2029
Annual dog vaccination coverage (%)	70%	20, 20, 20%		5, 5%			
Elimination target	WHO region	Estimated dog population (millions)	Total doses in millions (per year)								
2020	EURO (Eurasia)	85,612,000	300 (60, 60, 60, 60, 60)	51 (17, 17, 17)	351	8 (4, 4)	359	20.7	
	EMRO	26,547,000	95 (19, 19, 19, 19, 19)	15 (5, 5, 5)	110	2 (1, 1)	112	6.4	
	SEARO without India	72,631,000	255 (51, 51, 51, 51, 51)	45 (15, 15, 15)	300	8 (4, 4)	308	17.8	
	India (72% rural)	38,109,000	135 (27, 27, 27, 27, 27)	24 (8, 8, 8)	159	4 (2, 2)	163	9.4	
	WPRO without China	38,847,000	135 (27, 27, 27, 27, 27)	24 (8, 8, 8)	159	4 (2, 2)	163	9.4	
	China (55% rural)	71,785,000	250 (50, 50, 50, 50, 50)	42 (14, 14, 14)	292	8 (4, 4)	300	17.4	
Totals for EMRO, EURO, SEARO, and WPRO	333,531,000	1,170 (234 million doses a year)	201	1,371	34	1,405	81.2	1,405
2030	AFRO total	77,417,000	Program phase	Attack phase (5 years)	Consolidation phase (3 years)	Total (phases 1 and 2)	Maintenance phase		
				2020–2024	2025–2027	2020–2027	2028–2029		
				270 (54, 54, 54, 54, 54)	45 (15, 15, 15)	315	8 (4, 4)	18.9	328
Grand total WHO Regions		411,000,000						100	1,728

According to a 10-year national dog immunization program (assuming an attack phase of 5 years achieving a 70% coverage annually, consolidation phase of 3 years with 20% annual coverage and maintenance phase of 2 years with 5% annual coverage). AFRO, African Regional Office; EMRO, East Mediterranean Regional Office; EURO, European Regional Office; SEARO, South East Asia Regional Office; WHO, World Health Organization; WPRO, West Pacific Regional Office.

Based on these estimates, almost 1.3 billion doses (more than 260 million doses annually including a 10% wastage rate) are needed from 2015 to 2019 for the attack phase in Asia and the Middle East. This is a conservative figure, as human populations of urban centers of China and India, which have almost a billion people and associated large dog populations, were excluded from this computation. In India, a special rabies control program targets major cities ^88^. In large Chinese cities, dog rabies prevalence is lower, but dog ownership laws (though somewhat relaxed recently and often difficult to enforce) may remain quite strict ^89^. In addition, 560 million doses are required from 2020 to 2024 (about 110 million doses annually) for the consolidation phase in these areas to initiate and conduct programs in Africa and maintain freedom in previously liberated areas. This means providing during the period of 2015–2019, on an annual basis, five times more dog vaccine to rural India and China, three times more for Eurasia, and two times more than currently used for the Eastern Mediterranean Region as well as five times more to the entire of Africa during the period of 2020–2024. Given the other existing priority market requirements, quantities needed in the short term (2015–2019) are likely to exceed major manufacturers’ capacities of scaling-up. In addition, as almost 50% of these dog vaccine doses are needed in South, South Eastern, and Eastern Asian countries, especially in China and India, current procurement mechanisms for pure, potent, safe, and efficacious vaccines, mostly involving Northern Hemisphere manufacturers’ production units, will not be acceptable by all.

Besides mass dog vaccination, to achieve the concomitant goal of human rabies elimination by 2030, international organizations must also support RIG manufacturing (particularly in developing countries), fast-track the addition of new vaccines to the WHO list of pre-qualified rabies vaccines, and invest in production of new, safer, and more affordable biologics for passive immunization ^51, 78, 90^.

Together with national public health authorities, these organizations must strongly discourage the unnecessary administration of very large and inappropriate numbers of PEP, which contribute to creating shortages in areas where these rabies biologics are truly needed ( Table 8).

**Table 8.** Post-exposure prophylaxis (PEP), number and percentages of non-exposed persons receiving PEP per million.

Geographical cluster	World Health Organization region	Human populations	PEP per million people (Total PEP administered per cluster)	Exposures per million (Total exposures)	Number of non- exposed humans receiving PEP per million	Percentage of non-exposed humans receiving PEP
Asia						
Total Asia 2	WP and SEA	239,987,963, about 240 million	4,764 (1,143,377)	3,195 (766,842)	1,569	0.33
Total Asia 3	SEA	367,288,960, about 367 million	914 (335,740)	667 (244,767)	247	0.27
Total Asia 4	SEA and WP	185,887,076, about 186 million	4,638 (862,641)	1,413 (262,841)	3,225	0.70
Subtotal			2,341,758	(1,274,540)		
Total other Asia	Indonesia, China, India	2,859,385,227, about 2.8 billion	8,183 (23,395,261)	4,550 (13,008,455)	3,633	0.44
Subtotal			25,737,019	(14,282,905)		
Eurasia	EUR	898,926,561, about 899 million	748 (672,177)	289 (259,650)	495	0.66
Africa						
SADC	AFR	292,678,308, about 293 million	1,766 (517,409)	925 (271,041)	841	0.48
North Africa	EMR	20,982,504, about 21 million	1,917 (402,632)	930 (195,237)	987	0.51
West Africa	AFR	327,361,302, about 327 million	1,071 (350,374)	790 (258,341)	281	0.26
Other Africa	AFR	438,878,367, about 439 million	265 (116,433)	273 (119,707)	-8	-0.03
Subtotal			1,386,848	844,326		
EMRO					
	Minus North Africa and Somalia	251,146,263, about 251 million	932 (233,883)	465 (116,785)	467	0.50

Adapted from 70. AFR, African region; EMR, East Mediterranean region; EMRO, Eastern Mediterranean Regional Office; EUR, European (Asian portion) Region; SADC, Southern Africa Development Community; SEA, South East Asia; WHO, World Health Organization; WP, West Pacific.

Concerning dog rabies vaccines, their sustained availability at the ground level has repeatedly been shown to be essential to the initiation of dog rabies control activities ^85^. Essential steps to effectively increase veterinary rabies vaccine production to achieve human-dog-mediated rabies elimination by 2030 must be seriously considered by international organizations and vaccine manufacturers supporting this goal. These steps include accelerating technology transfer to Africa and Asia and redirecting some existing human rabies vaccine manufacturing capacities toward canine vaccine production, as proposed more than 30 years ago by a joint Rockefeller/WHO initiative ^91^. Similarly, GAVI support of human rabies vaccines might allow those eligible governments to use any conserved resources directed to veterinary public health, as begun regionally in the Americas in the 1980s. Comparatively modest investment in canine vaccination programs is predicted to have major cost-effective outcomes for the human rabies burden, even in some of the most severely affected areas ^92^.

## Future vision

Viral taxonomy may continue to adjust subjectively to changing times, differing philosophies, and bio-political pressure, yet regardless of the specific etiological names responsible for this disease, the moniker for rabies will remain. Theories on lyssavirus and host co-evolution will be complemented by methodological improvements and pathogen discovery. With enhanced, de-centralized laboratory-based surveillance and focused detection efforts, additional lyssavirus species and host shifts are expected. Stockpiling of vaccine and RIG, attention to existing recommendations, and careful risk assessments will increase the use of pre-exposure vaccination, maximize appropriate use of biologics in those truly exposed, and reduce PEP failures. Eventual application of the growing knowledge base on rabies pathogenetic mechanisms, pronounced intensive clinical skills in managing encephalitis, and the abilities to intervene with combinations of biologics and anti-viral drugs will gradually result in additional rabies survivors in human and veterinary medicine without losing the critical focus upon disease prevention. Ideally, under a progressive global business plan (with the minimum application of estimated biologics to the populations at risk, new champions, and dedicated sponsorships without vaccination fatigue), human rabies mediated by dogs should be eliminated in Latin America over the next 5 years, in Asia within a decade, and in Africa by 2030. Although eradication is not the aim, given a diversity of wildlife reservoirs and a lack of strategies to break the chain of perpetuation among the Chiroptera, the time is long overdue to accomplish such other ambitious goals against an ancient and insidious but neglected killer.
